# High-Precision Carton Detection Based on Adaptive Image Augmentation for Unmanned Cargo Handling Tasks

**DOI:** 10.3390/s24010012

**Published:** 2023-12-19

**Authors:** Bing Liang, Xin Wang, Wenhao Zhao, Xiaobang Wang

**Affiliations:** Naval Architecture and Ocean Engineering College, Dalian Maritime University, Dalian 116026, China; wx5201314@dlmu.edu.cn (X.W.); zwh1120221862@dlmu.edu.cn (W.Z.); wxb@dlmu.edu.cn (X.W.)

**Keywords:** cargo handling, data augmentation, target detection, YOLO

## Abstract

Unattended intelligent cargo handling is an important means to improve the efficiency and safety of port cargo trans-shipment, where high-precision carton detection is an unquestioned prerequisite. Therefore, this paper introduces an adaptive image augmentation method for high-precision carton detection. First, the imaging parameters of the images are clustered into various scenarios, and the imaging parameters and perspectives are adaptively adjusted to achieve the automatic augmenting and balancing of the carton dataset in each scenario, which reduces the interference of the scenarios on the carton detection precision. Then, the carton boundary features are extracted and stochastically sampled to synthesize new images, thus enhancing the detection performance of the trained model for dense cargo boundaries. Moreover, the weight function of the hyperparameters of the trained model is constructed to achieve their preferential crossover during genetic evolution to ensure the training efficiency of the augmented dataset. Finally, an intelligent cargo handling platform is developed and field experiments are conducted. The outcomes of the experiments reveal that the method attains a detection precision of 0.828. This technique significantly enhances the detection precision by 18.1% and 4.4% when compared to the baseline and other methods, which provides a reliable guarantee for intelligent cargo handling processes.

## 1. Introduction

The port is an important channel for import–export trade and economic growth. Cargo handling is the most time-consuming task during cargo trans-shipment and is a key factor leading to cargo backlogs and reduced port throughput. In addition, the virus can be easily transmitted during port operations. Therefore, unattended and intelligent cargo handling is key to improving the efficiency of port operations and reducing the rate of virus transmission. Since cargo is often packed in cartons, carton detection has become one of the core technologies in the intelligent cargo handling process [1,2].

**Figure 1:**
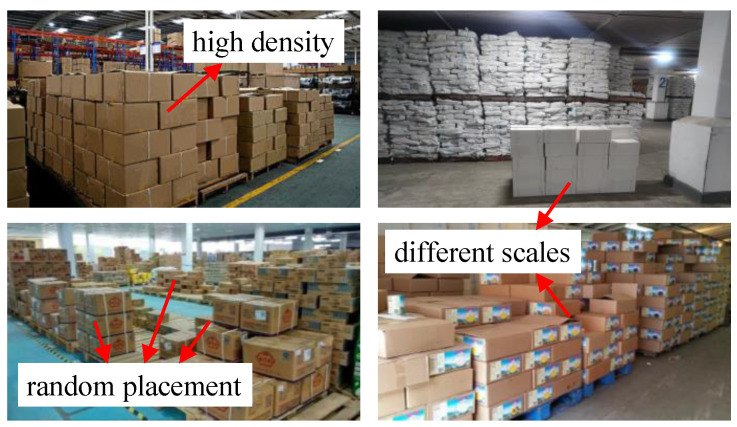
Samples of bulk cargo.

In the process of cargo handling, the intelligent cargo handling system needs to detect each carton in advance and generate corresponding grab instructions. Bulk cargo are characterized by high density, random placement and different scales, as shown in Figure 1, which seriously aggravates the difficulty of carton detection. In view of the high density and poor boundary discrimination of stacked cartons and the serious interference of other rectangular objects to carton detection, traditional image processing methods that are sensitive to environment have poor generalization and are not suitable for carton detection in intelligent cargo handling, while the object detection method based on deep learning has a strong generalization ability by relying on a large amount of data training and is widely used in carton detection. Therefore, this paper focuses on the deep-learning-based carton detection method and optimizes its performance in the carton detection process.

In this paper, a large-scale carton dataset is first presented to train the carton detection model, which includes the logistics cartons, containers and bulk cargo under various interference environments. Traditional deep learning algorithms, such as regions with convolutional neural network (R-CNN) [3] and You Only Look Once (YOLO) [4,5], need to be trained and learned on the basis of a wide range of datasets to improve model precision and generalization. However, in port operations scenarios, where the carton angle and imaging parameters have strong randomness, it is difficult for the presented carton dataset to cover all the cases. Combined with the high density and poor boundary discrimination of the stacked cartons, the trained model generalization ability is sharply reduced, resulting in a precision reduction of carton detection, multiple cartons being detected as one or even cartons not being detected. Therefore, it is particularly important to improve the generalization ability of the carton detection model based on the presented limited carton dataset.

It is time-consuming and impossible to collect the targets to be detected in all scenarios to solve the problem of a poor model generalization ability. Therefore, traditional deep learning methods augment the training sample set from the aspects of imaging parameters and perspective of a single image or synthesis of multiple images. Single image augmentation methods generate new images through style transfer [6,7], motion blur [8], perspective transformation [9], rotation [10], cropping [11], etc., while multi-image synthesis augmentation methods generate new images by pasting cropped foreground objects onto a new background [12,13,14]. However, there are still two main disadvantages of the traditional data augmentation methods when adopting them in carton detection: (1) traditional data augmentation methods only transform the images on the training set, but ignore the impacts of the actual environment on target detection, resulting in limited improvement in the model generalization ability; (2) for dense targets such as a carton stack, there are no purposeful augmentations on the indistinguishable target boundaries in traditional data augmentation methods, which still leads to a low precision of target detection.

To overcome the disadvantages of traditional deep learning methods in carton detection, this paper proposes a data augmentation method that takes into account the interferences of both the multiple scenarios and indistinguishable target boundaries. Firstly, since it is difficult for the presented training set to cover all the scenarios, an adaptive augmentation method for complementary scenarios is proposed, which transforms the background and perspective of the carton dataset to adapt to various practical scenarios. Then, aiming at the problem of the poor boundary discrimination of stacked cartons, a stochastic synthesis method of multiple boundary features is proposed to enhance the detection ability of deep learning methods to the boundary features. Finally, a hyperparameters optimization method of detection model based on an modified genetic algorithm (GA) is proposed to further improve the detection precision. Extensive experimental results on YOLO [15] demonstrate the effectiveness of the proposed method in improving the generalization ability of the carton detection model, and this method can better guide the intelligent cargo handling system to generate grab instructions.

The remainder of this paper is organized as follows. Section 2 reviews the previous work related to this paper. Section 3 details the proposed data augmentation method and the model hyperparameters optimization method. Experiments are presented in Section 4 and discussed in Section 5. Finally, the paper is concluded in Section 6.

## 2. Related Work

This paper is devoted to solving the problem of the generalization ability of a detection model. At present, there are two main approaches. (1) Deep learning networks are optimized to enhance the learning ability of the detection model. (2) Data augmentation strategies are proposed to realize the volume expansion of the limited data samples.

### 2.1. Deep Learning Models

According to the number of stages in the object recognition process, deep = learning-based methods for object recognition fall into two categories: two-stage series and one-stage series [16]. The two-stage series are first proposed, and the representative methods are R-CNN [17], Faster R-CNN, etc. Subsequently, to improve the efficiency of object recognition, one-stage series are proposed, which are represented by YOLO [18].

For the two-stage series, a Faster R-CNN model was proposed based on the R-CNN model with a precision of 45–79%, in which selective search was carried out first to determine the candidate area, and then target detection was performed to enhance the pertinence of detection [19]. Subsequently, a target feature extraction and detection model was proposed based on a Mask R-CNN, which improved the precision by 2.64% compared with the Faster R-CNN [20]. To overcome the problem of training set insufficiency, a Global Mask R-CNN detection algorithm based on a small training set was also presented by precisely composing the target feature region and saving the target semantic information in the deep learning backbone, and the precision could reach 66.45% [21]. For the one-stage series, the YOLOs are progressively proposed to improve the network structure, such as YOLO9000 [22], YOLOv3 [15] and YOLOv5 [23]. In one YOLOv3-based ship detection case, the detection precision could reach 55.3% [24]. By combining the CenterNet and YOLOv3 and introducing the spatial shuffle-group enhance (SSE) attention module, more advanced semantic features were integrated, avoiding the problem of detection omissions, and the precision was further improved to 90.6% [4]. On this basis, an extra detection head was added to the YOLOv5 model to improve the multi-scale detection and small target, experiencing an 11.6% rise [23]. In view of the better performance of YOLO series, this paper used YOLOv5 as the baseline to demonstrate the effectiveness of the proposed method.

### 2.2. Data Augmentations

There are two different approaches to data augmentation: transforming a single image and synthesizing multiple images. For the transformation of a single image, augmentation strategies such as color jittering [25], auto or rand augment [26,27], motion blur [8], perspective transformation [9,28], stochastic cropping [11,25,29] and rotation [10] can effectively improve the learning ability on the training set. However, these methods do not gain much regarding the generalization ability of the detection model because the training set is randomly transformed rather than according to the actual scenarios that can occur. In the aspect of multi-image synthesis, the cut-and-paste methods [12,30] are adopted. However, in synthetic images, the contextual semantic relationship between the target and the background is too stiff to effectively improve the precision of the detection model. The literature [12,14,31] hopes to improve the detection precision by ignoring the subtle pixel artifacts in the synthesized image, but the pixel artifacts are unavoidable [32].

### 2.3. Discussion

For the optimized deep learning networks, the precision of training sets is indeed greatly improved; however, the improvement effects on the prediction sets are not particularly evident, especially in the carton stack detection process, and there will still be a large number of detection omissions or errors. In comparison, data augmentation methods can effectively expand the training sets and improve the generalization ability of the detection models on the prediction sets. However, the interference of actual scenarios is not taken into account in the existing data enhancement method, which limits the precision of target detection in the actual scenarios. Therefore, a data augmentation method allowing for multiple scenarios and indistinguishable target boundaries is proposed in this paper.

## 3. Methodology

**Figure 2:**
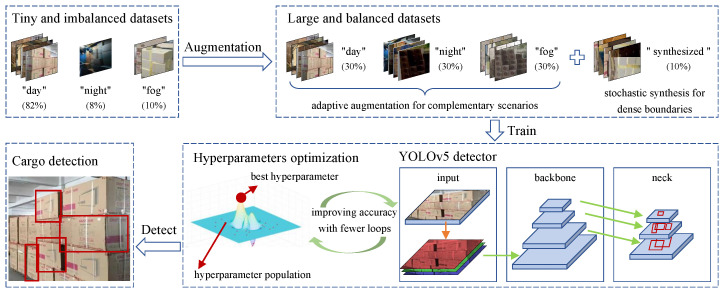
The complete process of our method.

The goal of the present study is three-fold. First, this study seeks to investigate the distribution law of imaging parameters in multiple scenarios and to construct matrices of imaging parameters in complementary scenarios for each specific scenario, thus enabling adaptive augmentation of complementary scenarios. Second, for problems where dense boundaries are indistinguishable, this study attempts to propose a stochastic synthesis method for multi-boundary features to enable boundary enhancement during training. Third, the correlation between model hyperparameters and model fitness is explored to improve the crossover probability function in GA, and the optimization of the model hyperparameters is achieved by the modified GA. The complete process of our method is shown in Figure 2.

### 3.1. Adaptive Augmentation for Complementary Scenarios

**Figure 3:**
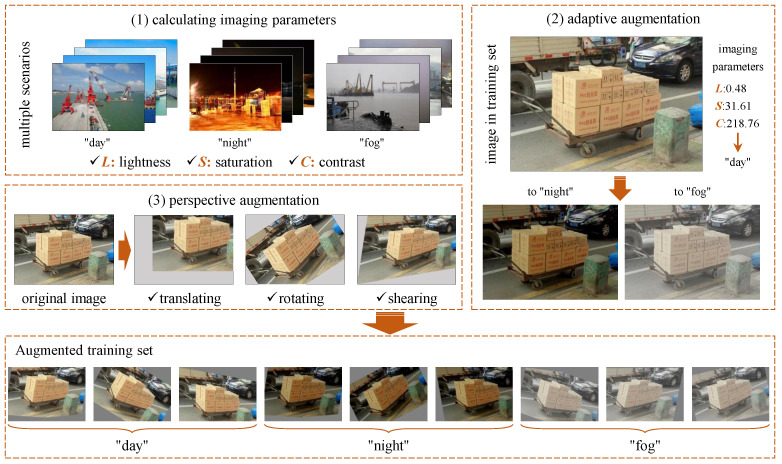
Architecture of the adaptive complementary augmentation approach.

The size of the training set and its coverage of the various scenarios determine to some extent the precision and generalization ability of the carton detection model. Since it is time-consuming and impossible to artificially collect carton samples in all the scenarios, this paper proposes an adaptive augmentation method for complementary scenarios based on carton samples in limited scenarios, which significantly reduces sample collection and labeling efforts. This approach involves three steps. (1) Calculation of imaging parameters: The imaging parameters in multiple scenarios, such as lightness, saturation and contrast, are calculated according to a large number of easily collected images in daytime, night, fog, etc. (2) Adaptive augmentation: New images are derived by converting the imaging parameters of each carton sample into the imaging parameters calculated above. (3) Perspective augmentation: Perspective augmentation is also applied to take into account the differences in the perspective of the cartons during the actual image acquisition. The architecture of this approach is shown in Figure 3.

The adaptive augmentation approach for complementary scenarios is detailed as follows.

First, the imaging parameters in multiple scenarios need to be calculated. Images from multiple scenarios are collected stochastically for imaging parameter calculation. For illustrative purposes, the scenarios are roughly classified as “day”, “night” and “fog”, and the imaging parameters of lightness, saturation and contrast are taken into account in this paper. Lightness *L*, saturation *S* and contrast *C* can be calculated through Equation (1).

(1) $$\left\{\begin{array}{cc} L & =\frac{1}{2}\left(MAX+MIN\right)\\ S & =\frac{MAX-MIN}{1-|2L-1|}\\ C & =\sum_{\delta }\delta {\left(u,v\right)}^{2}Pr_{\delta }\left(u,v\right) \end{array}\right.$$

where MAX and MIN are the maximum and minimum values of $\left(\bar{R},\bar{G},\bar{B}\right)$. $\left(\bar{R},\bar{G},\bar{B}\right)$ are the average values of the red (R), green (G) and blue (B) channels of an image, respectively. (u,v) represents the horizontal and vertical coordinates of a given pixel on an image, δ(u,v) is the gray level difference between the adjacent pixels and (u,v) and $Pr_{\delta }\left(u,v\right)$ is the distribution probability of the pixels with the gray level difference of δ.

The average value of the imaging parameters of the images from each scenario will be taken to represent the imaging parameters of the scenario, which can be expressed as:

(2) $${\bar{P}}_{sc}=\left({\bar{L}}_{sc},{\bar{S}}_{sc},{\bar{C}}_{sc}\right)$$

where ${\bar{P}}_{sc}$ is the imaging parameter representing the sc scenario (sc for day, night and fog), and ${\bar{L}}_{sc}$, ${\bar{S}}_{sc}$, ${\bar{C}}_{sc}$, respectively, stand for the lightness, saturation and contrast in ${\bar{P}}_{sc}$.

Then, for the *i*th image in the training set, the imaging parameter $P_{i}$ can also be calculated through Equation (1), which is denoted as:

(3) $$P_{i}=\left(L_{i},S_{i},C_{i}\right),i=\left(1,2,\ldots \right)$$

Proceeding to the next step, the new image will be generated by converting $P_{i}$ to ${\bar{P}}_{sc}$. $L_{i}$ and $C_{i}$ are converted by Equation (4).

(4) $$f_{i\_sc}^{\prime }\left(u,v\right)=\alpha f_{i}\left(u,v\right)+\beta$$

where $f_{i}\left(u,v\right)$ and $f_{i\_sc}^{\prime }\left(u,v\right)$, respectively, represent (R,G,B) on (u,v) of the *i*th image in the training set and its derived image. α is the contrast coefficient and β is the lightness gain coefficient.

After that, ${\bar{S}}_{sc}$ will be converted by Equation (5).

(5) $$s_{i\_sc}^{\prime }\left(u,v\right)=\left(1+\gamma \right)s_{i\_sc}\left(u,v\right)$$

where $s_{i\_sc}\left(u,v\right)=\frac{max\left(f^{\prime }\right)-min\left(f^{\prime }\right)}{1-|max\left(f^{\prime }\right)+min\left(f^{\prime }\right)-1|}$, $f^{\prime }$ is short for $f_{i\_sc}^{\prime }\left(u,v\right)$, $s_{i\_sc}^{\prime }\left(u,v\right)$ is the saturation of the newly derived image and γ is the saturation adjustment coefficient.

Thus, the *i*th image in the training set can be converted to a new image with the imaging parameters of ${\bar{P}}_{sc}$ through a set of appropriate coefficients of α, β and γ.

Finally, considering the effect of imaging perspective, perspective augmentation is implemented by translating, rotating and shearing an image according to Equations (6)–(8).

(6) $${\left(u^{\prime },v^{\prime }\right)}^{T}={\left(u,v\right)}^{T}+{\left(u_{t},v_{t}\right)}^{T}$$

(7) $${\left(u^{\prime },v^{\prime }\right)}^{T}=\left[\begin{array}{cc} cos\theta_{r} & sin\theta_{r}\\ sin\theta_{r} & cos\theta_{r} \end{array}\right]{\left(u,v\right)}^{T}$$

(8) $${\left(u^{\prime },v^{\prime }\right)}^{T}=\left[\begin{array}{cc} cos\phi_{u} & 0\\ sin\phi_{u} & 1 \end{array}\right]\left[\begin{array}{cc} 1 & sin\phi_{v}\\ 0 & cos\phi_{v} \end{array}\right]{\left(u,v\right)}^{T}$$

where $\left(u^{\prime },v^{\prime }\right)$ represents the transformed pixel coordinates after the original (u,v) transformation, $u_{t}$ and $v_{t}$ are the translations of (u,v) along the horizontal and vertical axes, respectively, $\theta_{r}$ is the rotation angle and $\phi_{u}$ and $\phi_{v}$ represent the shear angles along the horizontal and vertical axes.

The algorithm flow is shown in Algorithm 1.
**Algorithm 1** Adaptive Complementary Augmentation Algorithm**Input:** image sets of multiple scenarios Imsc, sc=(day,night,fog);             original training set Im;             allowable deviation of imaging parameters ϵ**Output:** augmented training set Imaug      1:**Initialize:** allowable error ϵ, α=1, β=0, γ=0, $\alpha_{r}=\left[\alpha_{rl},\alpha_{ru}\right]$, $\beta_{r}=\left[\beta_{rl},\beta_{ru}\right]$,$\gamma_{r}=\left[\gamma_{rl},\gamma_{ru}\right]$ are the searching range of α, β,γ      2:**# Imaging Parameters of Scenarios:**      3:**for** sc in (day,night,fog) **do**      4:      **for** image in Imsc **do**      5:            Calculate *L*, *S*, *C* for each image by Equation (1)      6:      Calculate ${\bar{P}}_{sc}=\left({\bar{L}}_{sc},{\bar{S}}_{sc},{\bar{C}}_{sc}\right)$ by Equation (2)      7:**# Appropriate Coefficients of α, β, γ:**      8:**for** *i*th image in Im **do**      9:      Calculate $P_{i}=\left(L_{i},S_{i},C_{i}\right)$ by Equation (3)    10:      **for** sc in (day,night,fog) **do**    11:            $\mathrm{err}={\bar{P}}_{sc}-P_{i}$    12:            **while** $\left|\mathrm{err}\right|>\epsilon {\bar{P}}_{sc}$ **do**    13:                $\left(bo_{L},bo_{S},bo_{C}\right)=BOOL\left(err>0\right)$    14:               $\alpha =\frac{\alpha +\alpha_{r}\left[bo_{C}\right]}{2}$    15:               $\beta =\frac{\beta +\beta_{r}\left[bo_{L}\right]}{2}$    16:               $\gamma =\frac{\gamma +\gamma_{r}\left[bo_{S}\right]}{2}$    17:               Generate a new image by Equations (4) and (5)    18:               Calculate $P_{i}^{\prime }$ by Equation (3)    19:               $\mathrm{err}={\bar{P}}_{sc}-P_{i}^{\prime }$    20:        Save the new image in Imaug    21:**# Perspective Augmentation:**    22:**for** image in Imaug **do**    23:     Random generation of $\left(u_{t},v_{t}\right)$, $\theta_{r}$, $\phi_{u}$, $\phi_{v}$    24:     Augment by Equations (6)–(8) and save to Imaug    25:ReturnImaug

### 3.2. Stochastic Synthesis of Multi-Boundary Features

After the adaptive augmentation for complementary scenarios of the training set, the enhancement of boundary features is also considered to improve the recognition precision for dense targets. A stochastic synthesis approach of multi-boundary features is proposed in this paper, which can improve the weight of boundary features without greatly expanding the training set.

**Figure 4:**
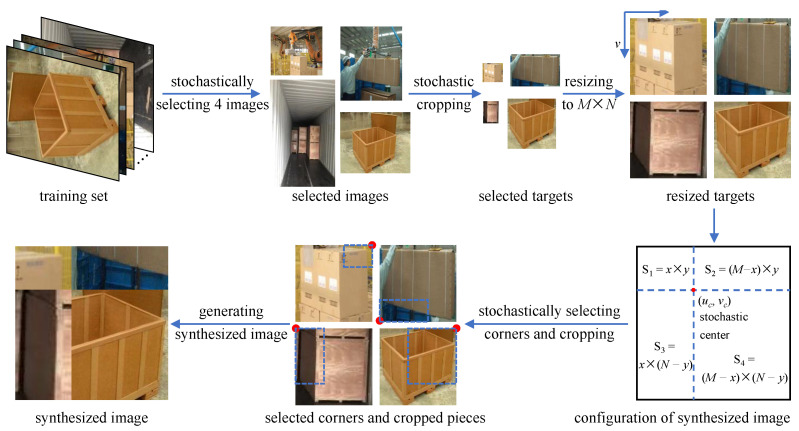
Flow of the stochastic synthesis approach.

The flow of the proposed approach is depicted in Figure 4. First, four images are selected stochastically from the training set to serve as metadata for a synthesized image. The targets in each image are then selected stochastically and cropped. To facilitate synthesis, cropped slices are resized to the size of the synthesized image. Meanwhile, a random center is generated to determine the configuration of the synthesized image. Then, a corner is chosen stochastically from the top left, top right, bottom left and bottom right in each resized cropped slice. Finally, the synthesized image is formed by image mosaics.

### 3.3. Hyperparameters Optimization Based on Modified GA

**Figure 5:**
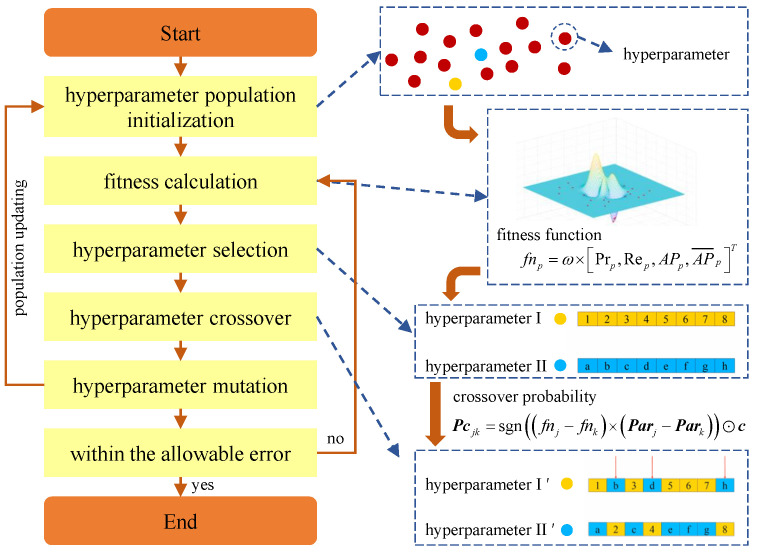
Hyperparameters optimization process based on the modified GA.

Based on adaptive complementary augmentation and boundary augmentation, the influence of model hyperparameters on the detection precision is also considered in this paper; thus, the GA is introduced to optimize the hyperparameters. However, in the existing GA, the stochastic crossover principle is employed in the gene crossover process with relatively low efficiency. As a result, a crossover probability function is developed to perform the optimal crossover of the genes and hence improve the optimization efficiency of the model hyperparameters. The hyperparameters optimization process is shown in Figure 5.

For illustrative purposes, population of model hyperparameters is generated stochastically in the hyperparameters ranges as follows.

(9) $$\mathrm{Par}={\left[{\mathrm{Par}}_{1},\ldots ,{\mathrm{Par}}_{p},\ldots ,{\mathrm{Par}}_{P}\right]}^{T}$$

where ${\mathrm{Par}}_{p}=\left[Par_{p1},\ldots ,Par_{pq},\ldots ,Par_{pQ}\right]$ represents the *p*th set of hyperparameters in the hyperparameters population ***Par***, p=(1,2,…,P), where *P* is the amount of the sets of hyperparameters, and q=(1,2,…,Q), where *Q* is the quantity of components in a set of hyperparameters; thus, $Par_{pq}$ represents the *q*th component in the *p*th set of hyperparameters.

To evaluate the model performance, four typical evaluation metrics are employed: (1) the precision Pr, (2) the recall Re, (3) the average precision AP for a specific value of the intersection over union (IoU) threshold to determine true positives (TPs) and false positives (FPs) and (4) the $\bar{AP}$, which averages AP across the different value of IoU thresholds from 0.5 to 0.95 with a step size of 0.05.

Then, a metric weight is set and a fitness function is established to simplify the evaluation process of the model performance, as shown in Equation (10).

(10) $$fn_{p}=\omega \times {\left[Pr_{p},Re_{p},AP_{p},{\bar{AP}}_{p}\right]}^{T}$$

where ω is the metric weight and $fn_{p}$ is the fitness of the model based on ${\mathrm{Par}}_{p}$. Thus, the fitness vector fn of the hyperparameter population Par can be expressed as:

(11) $$\mathrm{fn}={\left(fn_{1},\ldots ,fn_{p},\ldots ,fn_{P}\right)}^{T}$$

For a couple of selected hyperparameters from Par, component crossover will be performed to obtain a new set of hyperparameters. However, to achieve optimal crossover of hyperparameter components, the correlation of the model fitness with each component in the hyperparameters should be determined first, in which the statistical distributions of fn and Par with respect to their respective medians are employed. Thus, the correlation function is described as Equation (12).

(12) $$c_{q}={\left({\mathrm{Par}}_{∼q}-{\overset{ˇ}{\mathrm{Par}}}_{∼q}\right)}^{T}\times \left(\mathrm{fn}-\overset{ˇ}{\mathrm{fn}}\right)$$

where ${\mathrm{Par}}_{∼q}$ consists of the *q*th components in each ${\mathrm{Par}}_{p}$, $\overset{ˇ}{\left(\cdot \right)}$ represents the median value of (·) and $c_{q}$ is the correlation of the model fitness with the *q*th component, of which the positivity and negativity indicate the positive and negative correlations, respectively, and the absolute value reflects the correlation degree. Thus, the correlation vector ***c*** can be further expressed as:

(13) $$c=(c_{1},\ldots ,c_{q},\ldots ,c_{Q})$$

Furthermore, for a couple of hyperparameters, such as ${\mathrm{Par}}_{j}$ and ${\mathrm{Par}}_{k},j,k\in \left(1,2,\ldots ,P\right)$, the crossover probability function is established as:

(14) $${\mathrm{Pc}}_{jk}=sgn\left(\left(fn_{j}-fn_{k}\right)\times \left({\mathrm{Par}}_{j}-{\mathrm{Par}}_{k}\right)\right)⊙c$$

where ${\mathrm{Pc}}_{jk}$ is the crossover probability vector of each component in ${\mathrm{Par}}_{j}$ and ${\mathrm{Par}}_{k}$, sgn(·) represents the signum function, which is equal to +1 or −1, respectively, when (·)>0 or (·)<0, and ⊙ represents the bitwise multiplication of two vectors. Thus, new sets of hyperparameters can be obtained by crossover of ${\mathrm{Par}}_{j}$ and ${\mathrm{Par}}_{k}$ according to ${\mathrm{Pc}}_{jk}$. Finally, the optimal set of hyperparameters can be efficiently solved by the modified GA based on the introduced crossover probabilities. The algorithm flow is shown in Algorithm 2.
**Algorithm 2** Hyperparameters Optimization Algorithm**Input:** hyperparameters population ***Par*****Output:** the optimal set of hyperparameters Parop      1:**Initialize:** allowable error ϵ, the metric weight ω      2:**# Fitness Calculation:**      3:Calculate metrics $\left[Pr,Re,AP,\bar{AP}\right]$      4:Calculate fitness based on each ${\mathrm{Par}}_{p}$ in ***Par*** by Equation (10), and work out fitness vector ***fn*** by Equation (11)      5:**# Selection, Crossover and Mutation:**      6:**while** max(fn)−min(fn)>ϵ **do**      7:     Select: ${\mathrm{Par}}_{j}$, ${\mathrm{Par}}_{k}$ in ***Par***      8:     **for** ${\mathrm{Par}}_{∼q}$ in ***Par*** **do**      9:           Calculate $c_{q}$ by Equation (12)     10:     Calculate ${\mathrm{Pc}}_{jk}$ by Equation (14)     11:     Crossover: ${\mathrm{Par}}_{j}$, ${\mathrm{Par}}_{k}$⇒${\mathrm{Par}}_{jn}$, ${\mathrm{Par}}_{kn}$     12:     Mutation: stochastics and low probability     13:     Calculate fn based on ${\mathrm{Par}}_{jn}$ and ${\mathrm{Par}}_{kn}$     14:     **if** $fn_{jn}\;or\;fn_{kn}>min\left(\mathrm{fn}\right)$ **then**     15:          Remove min(fn), ${\mathrm{Par}}_{min}$     16:          Add $fn_{jn}\;or\;fn_{kn}$, ${\mathrm{Par}}_{jn}$ or ${\mathrm{Par}}_{kn}$     17:${\mathrm{Return}\mathrm{Par}}_{op}$ in ***Par*** with the maximum fitness

## 4. Experiments

This chapter mainly explores the application of adaptive complementary augmentation and stochastic synthesis approaches in the domain of carton training set expansion, as well as the role of the hyperparameters optimization method in improving the generalization ability of trained models. The effectiveness of our approaches is explored on YOLOv5, while the experiments are based on PyTorch 3.10 and performed on RTX3090.

### 4.1. Experimental Settings

**Figure 6:**
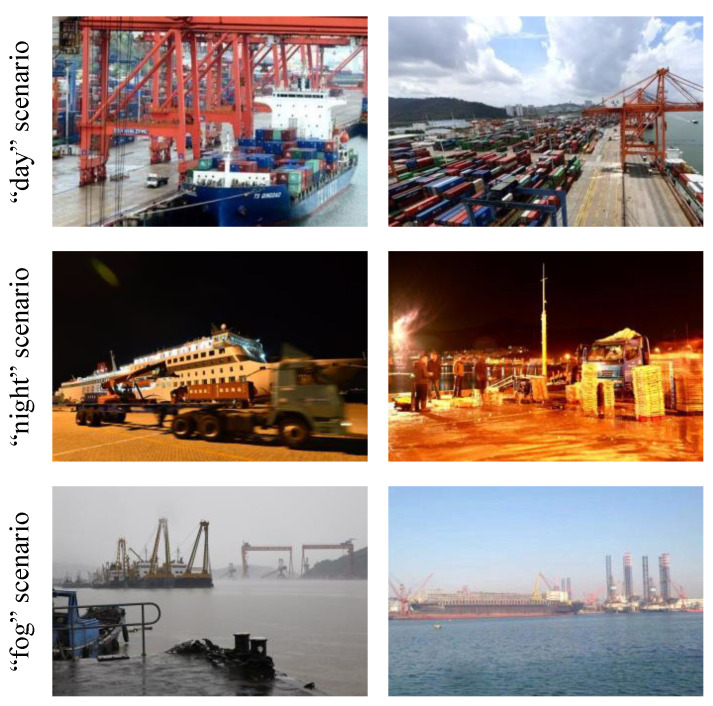
Samples of multiple-scenarios dataset.

**Multiple scenarios dataset** Since the imaging parameters of images in various scenarios are necessary for the adaptive complementary augmentation method, 200 images of ports or waters were collected for each scenario. Some samples are shown in Figure 6. Thus, the imaging parameters of each scenario can be calculated by Equations (1) and (2).

**Figure 7:**
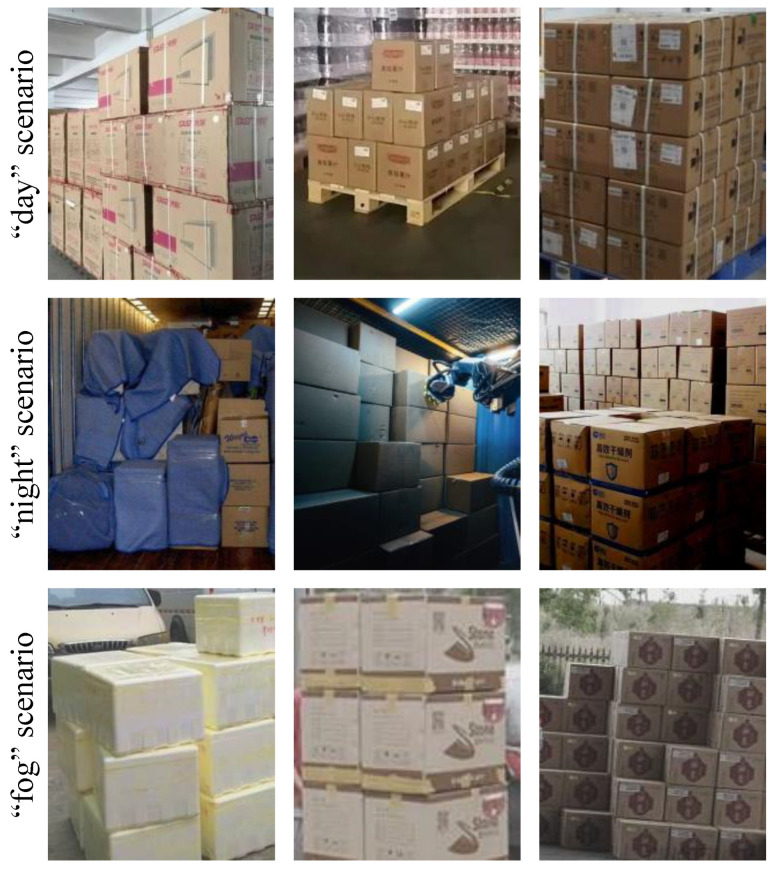
Samples of carton dataset.

**Carton dataset** The carton dataset in this paper refers to the stacked carton dataset (SCD) [33]. However, as a direct application of the proposed method on SCD is too time-consuming due to the large scale of the SCD, a portion of the sample is drawn from the SCD to form our carton dataset. The distribution of our carton dataset is given in Table 1. Due to the different difficulties in image collection under various scenarios, the images in the carton dataset are mainly collected under the “day” scenario, accounting for 81.7%, while the images collected under the “night” scenario and “fog” scenario only account for 8.2% and 10.1% respectively, resulting in a great reduction in the generalization ability of the trained model. Moreover, Figure 7 shows that cartons of different sizes are densely stacked and suffer from poor boundary discrimination, which severely affects the detection precision of cartons. Therefore, during the experiments, the carton dataset was split into a training set of 850 images and a testing set of 150 images, and the training set was augmented using the methods described in Section 3.1 and Section 3.2.

**Evaluation metric** Same as in Section 3.3, four typical evaluation metrics are employed: the precision Pr, the recall Re, the average precision AP when the IoU threshold is equal to 0.5 (denoted as AP@0.5) and the $\bar{AP}$, which averages AP across the different values of IoU thresholds from 0.5 to 0.95 with a step size of 0.05.

### 4.2. Adaptive Complementary Augmentation

**Figure 8:**
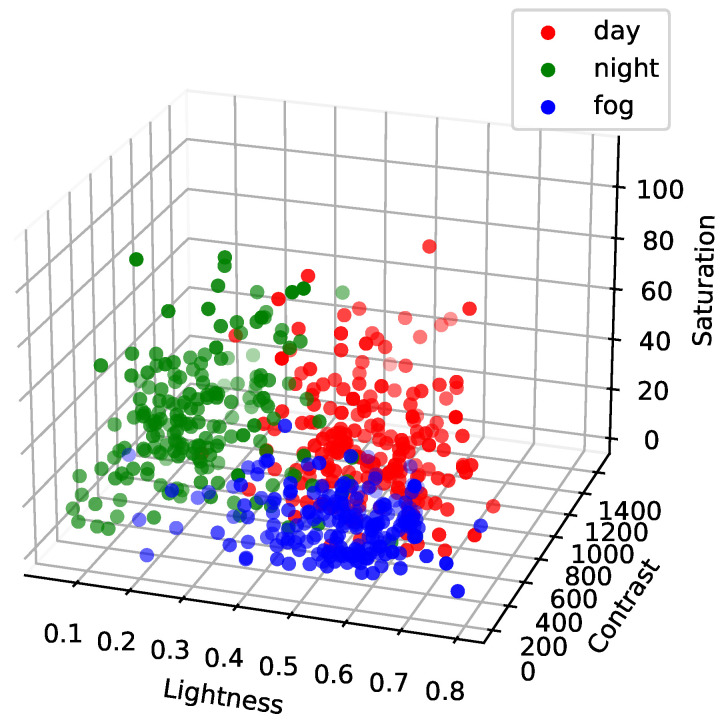
Distribution of imaging parameters.

Before performing augmentation for the training set, the imaging parameters were first calculated based on the multiple-scenarios dataset. The distribution of imaging parameters of each image in multiple scenarios is shown in Figure 8.

Figure 8 shows that the imaging parameters are obviously differentiated for different scenarios. Therefore, the average value of the imaging parameters in each scenario was taken to represent the imaging parameters in this scenario as follows.

(15) $$\left\{\begin{array}{c} {\bar{P}}_{day}=\left(0.562,409.447,39.678\right)\\ {\bar{P}}_{night}=\left(0.274,254.860,46.044\right)\\ {\bar{P}}_{fog}=\left(0.536,195.848,16.624\right) \end{array}\right.$$

**Figure 9:**
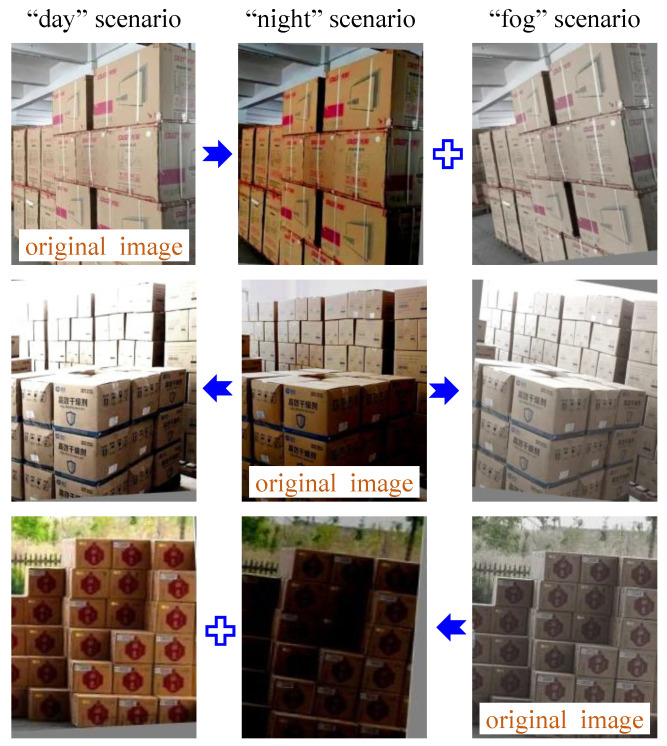
Samples of augmented images in training set.

Then, the imaging parameters of each image in the training set were calculated, based on which the images were classified into their corresponding scenarios. Then, following the adaptive complementary augmentation approach in Section 3.1, the imaging parameters of each image in the original training set were adjusted to those representing other scenarios. Further, two perspective augmentation methods were randomly selected from the translation, rotation and shear with two random conversion amplitudes. In this way, new images were generated as shown in Figure 9 and the training set was augmented.

**Figure 10:**
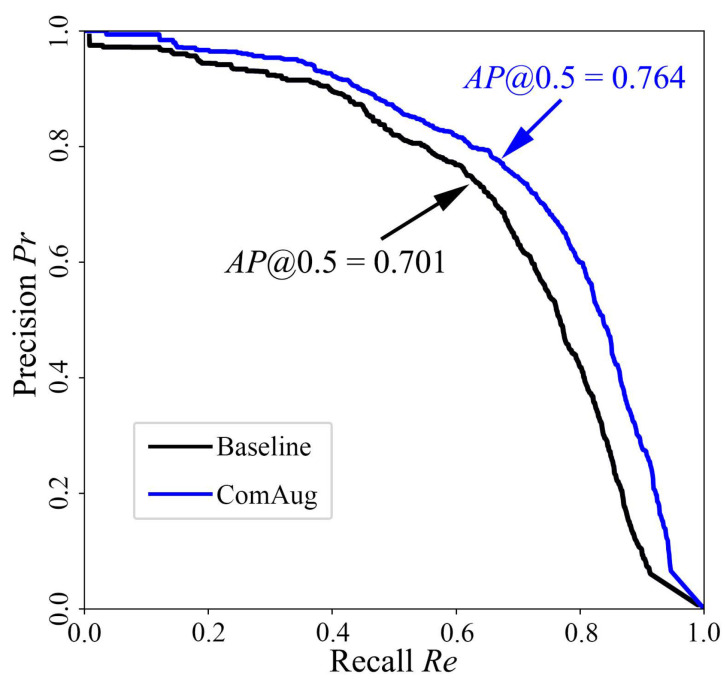
Precision–recall curve based on the original training set (Baseline) and the adaptive complementary augmented training set (ComAug).

Finally, the precision of the trained models based on the original training set and the augmented training set are compared in Figure 10. It can be seen that the adaptive complementary augmentation approach can effectively improve the model average precision AP@0.5 by 8.99% from the original 0.701 to 0.764.

### 4.3. Stochastic Synthesis

**Figure 11:**
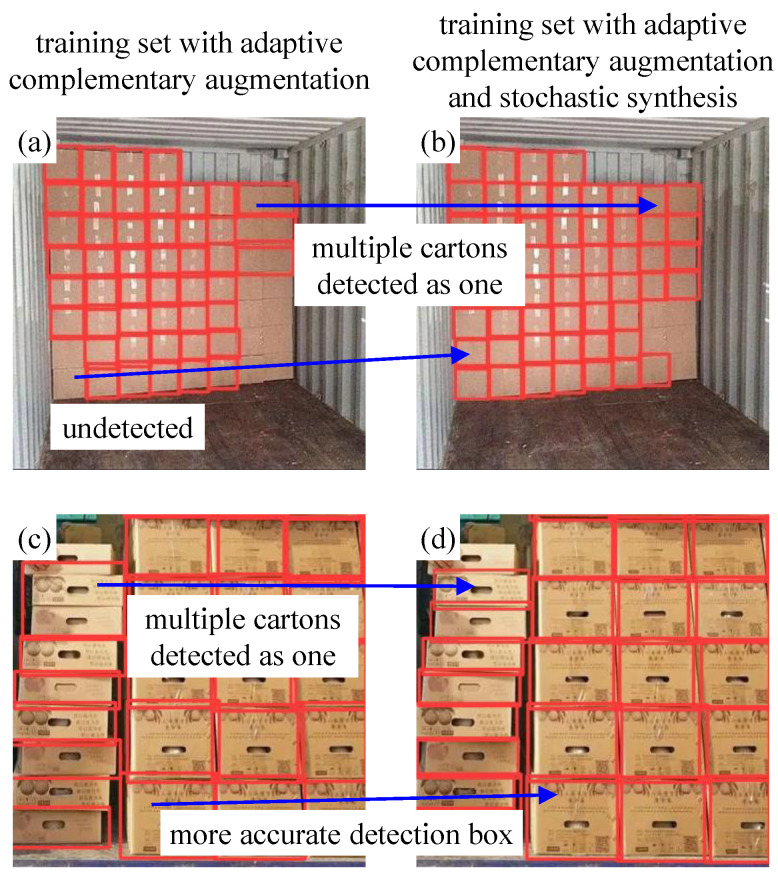
False detection of carton stack.

When using a model trained on a dataset without the synthesized images for detection, multiple cartons are easily identified as one due to the poor boundary discrimination of dense cartons, as shown in Figure 11a,c.

**Figure 12:**
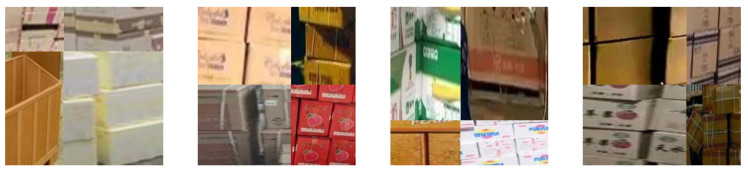
Some of the stochastic-synthesized images.

Therefore, the stochastic synthesis method in Section 3.2 was employed, and some of the stochastic-synthesized images are shown in Figure 12. The synthesized images enhanced the detection capability of the newly trained model on dense cartons, as shown in Figure 11b,d. It can be seen that, after the introduction of stochastic synthesis, cartons with indistinguishable boundaries can be detected separately, and previously undetectable ones can also be detected. At the same time, the detection box of each carton is more accurate due to the enhanced boundary features. Thus, the model average precision AP@0.5 is further improved by 3.80%, from 0.764 to 0.793.

### 4.4. Hyperparameters Optimization

Since model hyperparameters have an important impact on the precision of the trained model, it is necessary to perform a hyperparameters optimization process. However, due to the large expansion of the training set by the augmentation approaches proposed in this paper, even a single training procedure takes a long time. The hyperparameters optimization process based on the conventional GA can be time-consuming and requires a large number of iterations. Therefore, the modified GA in Section 3.3 is used to reduce the number of training iterations and significantly shorten the hyperparameters optimization time.

**Figure 13:**
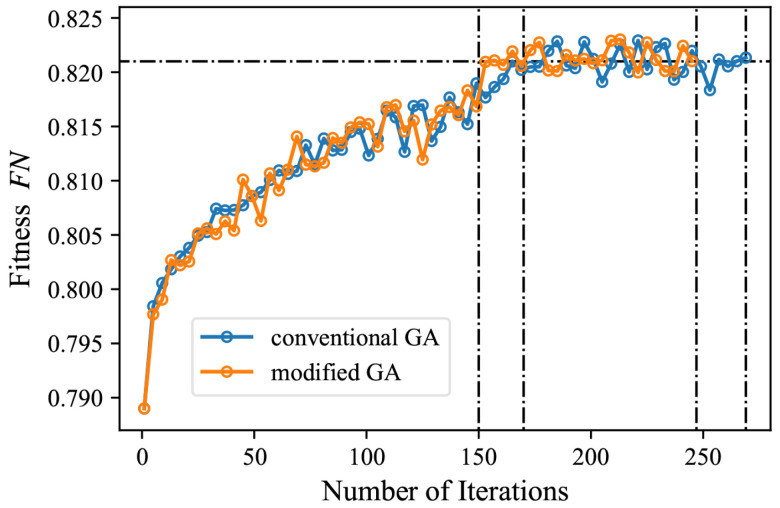
Hyperparameters optimization process based on conventional and modified GA.

With the FN=max(fn) in Equation (11) as the simplified evaluation of the trained model, Figure 13 shows the variation trend of fn during hyperparameters optimization when conventional and modified GAs are adopted. We observed that the hyperparameters optimization process based on the modified GA requires fewer iterations, resulting in an 8.9% reduction in time consumption.

### 4.5. Analysis of Carton Detection Precision

**Figure 14:**
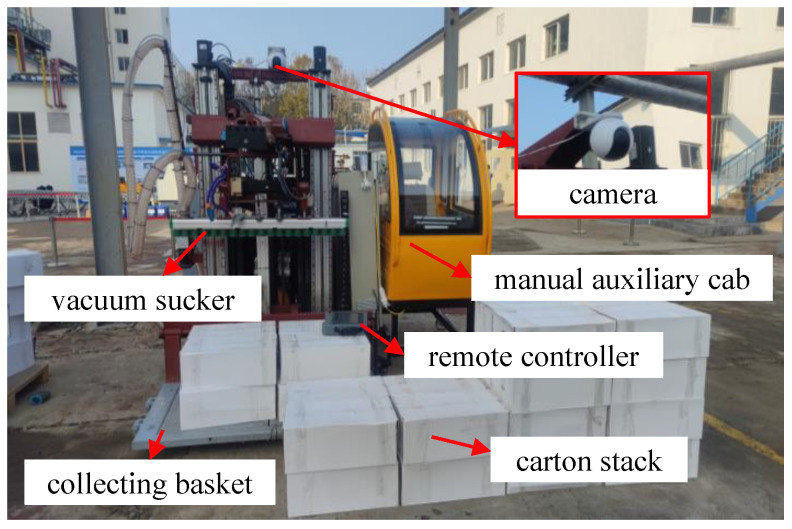
Intelligent cargo handling system.

**Figure 15:**
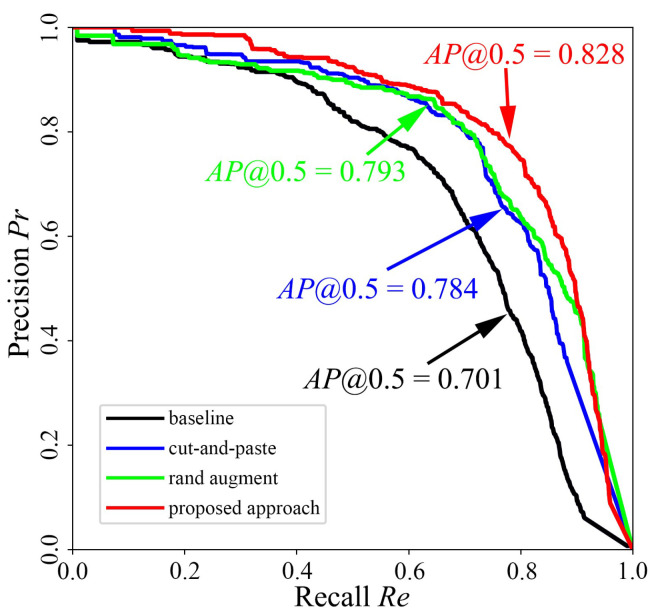
Precision–recall curve of carton detection.

To illustrate the effectiveness of the proposed approach, an intelligent cargo handling system has been designed as described in Figure 14. The evaluation metrics for the trained models of the proposed approach have been calculated using the images collected during the actual cargo handling process, and the comparison results among the alternative approaches are presented in Figure 15 and Table 2.

It can be seen that, with the introduction of the approach proposed in this paper, the precision, recall and other metrics of the trained model are greatly improved, and the average precision is increased by 18.1% from the initial 0.701 to 0.828, providing a good guarantee for the carton detection in the cargo handling process.

## 5. Discussion

The proposed method enables the automatic augmentation and balancing of images collected in various scenarios. Figure 7 illustrates the obtained images in different scenarios. As can be seen from Figure 7, the parameters of the images, such as brightness, saturation and contrast, vary considerably in different scenarios, as demonstrated in Figure 8. Through clustering analysis, the imaging parameters in each scenario are represented by the mean values of parameters such as brightness, saturation and contrast, which are used to guide the augmentation process of the collected images in each scenario, thus increasing the scale of the original dataset from 1000 to 3000, as shown in Figure 9. Figure 10 proves that the precision of the trained model on the augmented dataset is significantly improved compared to the baseline. However, the detection precision of dense boundaries still needs to be improved, as shown in Figure 11a,c. Thus, the boundary feature stochastic synthesis strategy is adopted to further augment the dataset scale from 3000 to 4000, which significantly improves the detection ability of the trained model, as shown in Figure 11b,d. To address the problem of decreasing training efficiency due to the large number of augmented datasets, Figure 13 demonstrates the effect of the modified GA. Compared to the traditional GA, the number of training iterations is reduced from 269 to 246, saving nearly 8.5%. Finally, Figure 15 compares the detection precision of the baseline, cut-and-paste, rand augment and the proposed method in the actual cargo handling process. As shown in Figure 15, the proposed method performs significantly better when compared to the other methods and the baseline, achieving a precision of 0.828 and an improvement from 4.4% to 18.1%.

In summary, we believe that our study contributes significantly to the recognition of dense objects in complex environments due to the simultaneous consideration of the complexity of the scenario, the poor boundary discrimination of the objects and the optimization of the model hyperparameters. The proposed adaptive augmentation method can balance the dataset, making the performance of the trained model better and more stable in each scenario. Meanwhile, the proposed stochastic synthesis method can overcome the effect of dense boundaries and improve the recognition precision. Moreover, with the proposed hyperparameter optimization method, the effect of the augmented dataset on the training speed is eliminated and the training efficiency is improved.

However, the proposed method still suffers from some shortcomings. In the actual cargo handling process, it is found that the proposed method has a significant effect on the detection precision for images collected in “night” and “fog” scenarios, but it is almost ineffective for images collected in the “day” scenario. The reason is that the images from the “day” scenario make up the majority of the original dataset; however, high quality datasets should be balanced. The method in this paper focuses on the balanced augmentation of datasets and is therefore beneficial for scenarios other than “day”. From a generalization point of view, for round-the-clock target detection efforts, there will be an inevitable imbalance in the dataset. Therefore, the method in this paper still has an important role and significance.

## 6. Conclusions

Carton detection is crucial for unattended intelligent cargo handling to achieve efficient port operations and reduce the virus transmission rate. However, cargo handling scenarios are diverse, and the carton stacks are characterized by high densities with indistinguishable boundaries. Therefore, this paper proposes a novel data augmentation approach to achieve a high detection precision, which takes into account the interferences of multiple scenarios and indistinguishable target boundaries. First, the distribution law of the imaging parameters in multiple scenarios is investigated, and the imaging parameters of each image in the training set are adjusted to those of the complementary scenario of that image, thus enabling adaptive augmentation of complementary scenarios. Then, the images in the training set are stochastically selected, cropped and synthesized to enhance the carton boundary features. Finally, the hyperparameters are also optimized through a modified GA to further improve the precision of the trained model. With the proposed approach, the trained model achieves a large improvement in average precision from 0.701 to 0.828 in the actual cargo detection process. Comparisons with other data augmentation methods are also performed to demonstrate the better performance of the proposed approach.

## Figures and Tables

**Table 1:** Distribution of the carton dataset.

Scenario	Carton Dataset	Training Set	Testing Set
“day”	817 (81.7%)	694	123
“night”	82 (8.2%)	70	12
“fog”	101 (10.1%)	86	15
ALL	1000	850	150

**Table 2:** Metrics comparison of trained models.

Approaches	Pr	Re	AP	$\bar{\mathrm{AP}}$
baseline	0.715	0.657	0.701	0.430
cut-and-paste [28]	0.732	0.714	0.784	0.460
rand augment [25]	0.741	0.719	0.793	0.493
proposed approach	**0.775**	**0.773**	**0.828**	**0.521**

## Data Availability

Data are contained within the article.
